# Intraperitoneal recurrence of renal haemangiopericytoma

**DOI:** 10.11604/pamj.2017.27.264.10106

**Published:** 2017-08-09

**Authors:** Fatima Zahra El M’rabet, Meryem Azegrar

**Affiliations:** 1 Department of Medical Oncology, University Hospital Hassan II, Fes, Morocco

**Keywords:** Haemangiopericytoma, recurrence, kidney

## Image in medicine

We describe a case of a 53 years old patient who underwent right nephrectomy for a renal tumor in 2005. The histological examination revealed an hemangiopericytoma with graveness signs. Ten years later, the patient developed a huge abdominal mass. CT scan showed a voluminous locally advanced tumor measuring 30*20 cm. The mass was multilobed and hypervascularized (A) Witch was compatible with a hemangiopericytoma. The biopsy confirmed the recurrence of the hemangiopericytoma (B). The patient received a palliative chemotherapy taking into consideration local and metastatic extension. Hemangiopericytoma is a rare vascular tumor that emerges from Zimmerman perocytes. Usually it’s a voluminous asymptomatic mass with variable malignancy potential, and no specified clinical or radiological aspects. The standard of care is surgery and it should be suggested even for recurrences, the management of locally advanced and metastatic disease consists on palliative chemotherapy for sarcoma and should be discussed in a pluridisciplinary reunion.

**Figure 1:**
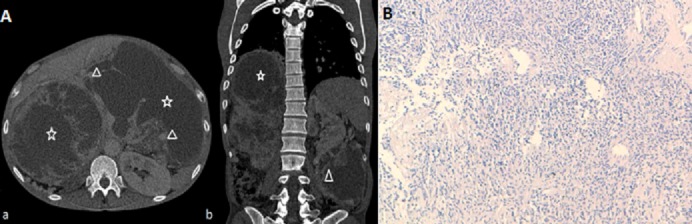
Axial (a) and coronal (b) scannographic cuts shouirng an abdominal solidcystic tumor (star) occupying the right nephrectomy space and peritoneum: the mass features a significant angiogenessis in portal phase (A); (B) variable proliferation density of spindle shaped cells disposing in short fascisles within a collagenos matrix; the vascularization is hemangiopericystic (HES X 20)

